# The role of SELE gene polymorphism in ST-elevation myocardial infarction

**DOI:** 10.18699/vjgb-25-16

**Published:** 2025-02

**Authors:** N.P. Babushkina, A.M. Nikolaeva, A.D. Dolbnya, V.E. Shavrak, V.V. Ryabov

**Affiliations:** Research Institute of Medical Genetics, Tomsk National Research Medical Center of the Russian Academy of Sciences, Tomsk, Russia; Cardiology Research Institute, Tomsk National Research Medical Center of the Russian Academy of Sciences, Tomsk, Russia; Siberian State Medical University of the Ministry of Healthcare of the Russian Federation, Tomsk, Russia; Tomsk State University, Tomsk, Russia; Cardiology Research Institute, Tomsk National Research Medical Center of the Russian Academy of Sciences, Tomsk, Russia Siberian State Medical University of the Ministry of Healthcare of the Russian Federation, Tomsk, Russia Tomsk State University, Tomsk, Russia

**Keywords:** ST-elevation myocardial infarction, STEMI, SELE gene, SNP, инфаркт миокарда с подъемом сегмента ST, ИМпST, ген SELE, SNP

## Abstract

Ischemic heart disease (IHD) is an important medical and social problem. ST-elevation myocardial infarction (STEMI) is the most severe form of IHD, affecting all layers of the heart muscle. One of the diagnostic criteria for endothelial dysfunction in myocardial infarction is the level of sE-selectin, a cell adhesion molecule that recruits neutrophils and induces neutrophil inflammation. The aim of this study is to investigate intronic polymorphisms rs5353, rs3917412 and rs1534904 of the E-selectin coding gene SELE in patients with STEMI. We have analyzed a group of patients with STEMI (n = 74) and a population sample of Tomsk (n = 136) as the control group. The frequencies of the rs5353 genotypes in the SELE gene have shown statistically significant differences between patients and the control sample (p = 0.004). The CC genotype is a predisposing factor to STEMI (OR = 6.93, CI:95 % (1.84–26.04), χ2 = 8.69, p = 0.002). The analyzed markers were not studied previously in cardiovascular diseases (CVDs) and were rarely involved in association studies at all; there is no information on these SNPs in the leading databases. At the same time, all three variants, according to the RegulomeDB classification, belong to the functional class 1f, and are highly likely to have regulatory potential relative not only to the SELE gene, but also to other genes in the nearby region. The analysis of the functional significance of the studied markers has shown the presence of a region more extensive than one gene, which is co-regulated by the studied nucleotide substitutions. The association of rs5353 with STEMI identified in this study once again confirms the involvement of the SELE gene in the pathogenesis of CVDs. It is possible that this entire region of the genome may be involved indirectly in the pathogenesis of CVD through the systems of inflammation, immune response and DNA repair.

## Abstract (ru)

Ишемическая болезнь сердца представляет собой важную медико-социальную проблему. Наиболее тяжелой формой заболевания, с поражением всех слоев сердечной мышцы, считается инфаркт миокарда с подъемом сегмента ST (ИМпST). Одним из диагностических критериев дисфункции эндотелия при инфаркте миокарда является уровень sE-селектина – молекулы клеточной адгезии, осуществляющей рекрутинг нейтрофилов и индукцию нейтрофильного воспаления. В настоящем исследовании изучен интронный полиморфизм (rs5353, rs3917412, rs1534904) гена SELE, кодирующего Е-селектин, у пациентов с ИМпST. Проанализированы две выборки: пациенты с ИМпST (n = 74) и популяционная выборка г. Томска (n = 136). По частотам генотипов rs5353 в гене SELE зарегистрированы статистически значимые различия между пациентами и контрольной выборкой (p = 0.004). Генотип СС является рисковым по отношению к ИМпST (OR = 6.93, CI:95 % (1.84–26.04), χ2 = 8.69, p = 0.002). Проанализированные маркеры не изучались ранее при сердечно-сосудистых заболеваниях и вообще редко привлекались к ассоциативным исследованиям; в ведущих базах данных отсутствует информация об ассоциациях этих маркеров с заболеваниями. Вместе с тем все три варианта по классификации RegulomeDB относятся к функциональному классу 1f и, соответственно, с высокой вероятностью обладают регуляторным потенциалом относительно не только гена SELE, но и других генов близлежащего региона. Анализ функциональной значимости изученных маркеров показал наличие более обширного, чем один ген, региона, корегулируемого данными нуклеотидными заменами. Выявленная в настоящем исследовании ассоциация rs5353 с ИМпST еще раз подтверждает вовлеченность гена SELE в развитие сердечно-сосудистых заболеваний. Не исключено, что опосредованно (через системы воспаления, иммунного ответа и репарации ДНК) весь этот регион генома может быть вовлечен в патогенез сердечно-сосудистых заболеваний.

## Introduction

Ischemic heart disease (IHD) is an important medical and social problem, holding the leading place in the structure of mortality from cardiovascular diseases. The most life-threatening condition is the acute form of ischemia, myocardial infarction. ST-elevation myocardial infarction (STEMI) is the most severe form with damage to all layers of the heart muscle (Clinical practice guidelines…, 2020). Inflammation is one of the leading elements in the pathogenesis, course and prognosis of myocardial infarction (Kachkovsky, Ragozina, 2013; Kalinin et al., 2022; Zhang N. et al., 2022). The inflammatory response is initiated by endothelial dysfunction associated with an imbalance in the production of endothelial mediators and leading to overexpression of adhesion molecules (Kachkovsky, Ragozina, 2013; Habas, Shang, 2018; Mathur et al., 2023).

E-selectin is a surface glycoprotein, that belongs to the class of cell adhesion molecules. E-selectin is expressed only by endothelial cells and exists in two forms: a transmembrane glycoprotein and a serum sE-selectin. In endothelium that is functioning normally, the amount of the protein is so low as to be negligible. E-selectin plays a role in the adhesion of neutrophils from circulating blood to the damaged vascular wall, and also promotes the migration of monocytes into the subendothelial space (Lorenzon et al., 1998; Vestweber, Blanks, 1999; Cid et al., 2000; Blankenberg et al., 2003; Calder et al., 2013; McEver, 2015)). In addition, the mechanism of neutrophil inflammation activation induced by E-selectin (through NLRP3 inflammasome activation) has been demonstrated (Pruenster et al., 2023). Given that neutrophils are the initial cells to infiltrate the site of damage during myocardial infarction (Kalinin et al., 2022), the pathogenetic role of E- selectin, which plays a dual role in the response to damage (neutrophil recruitment and induction of neutrophil inflammation), appears to be even more significant

De novo synthesis of E-selectin is initiated in the endothelium following stimulation with proinflammatory cytokines (TNF-α, IL-1), endotoxin, or under conditions of shear stress. Following initial exposure to the stimulus, the protein level rises within four to six hours, subsequently declining after one to two days. Therefore, E-selectin expression may reflect the acute phase of inflammation (Kalinin et al., 2022; Uy et al., 2024). Selectins in general, and E-selectin in particular, are well-recognized markers of endothelial dysfunction (Silva et al., 2018; Mangoni, Zinellu, 2024; Wang K. et al., 2024). The measurement of sE-selectin levels is a diagnostic tool used to diagnose endothelial dysfunction in patients with heart failure, atherosclerosis, glaucoma, DM2, arterial hypertension, ACS, an indicator of myocardial damage in children with respiratory mycoplasmosis (Mycoplasma pneumoniae), COVID-19, and other conditions (Wang N. et al., 2001; Ueno, 2012; Sandoval- Pinto et al., 2014; Srivastava et al., 2018; Lampsas et al., 2022; Mathur et al., 2023). There is evidence to support the hypothesis that E-selectin levels are associated with the presence of atherosclerotic vascular lesions, both coronary and peripheral (Zhito et al., 2019; Kalinin et al., 2022; Mathur et al., 2023). This is likely to reflect systemic inflammation as a characteristic feature of atherosclerosis.

The role of selectins in the pathogenesis of IHD is controversial. As is the case with numerous association studies, the accumulated evidence is contradictory: in some cases, authors have reported a statistically significant increase in E-selectin levels in patients with sTable IHD, whereas in other cases, no significant differences have been observed (see review (Zhito et al., 2019)). These results are explained by small sample sizes, heterogeneity in sex, age, presence of comorbidities, and the treatment received by patients (Zhito et al., 2019).

It is noteworthy that, despite a considerable amount of information dedicated to E-selectin, the focus is significantly shifted towards biochemistry: the protein level is analyzed in various pathological conditions and its role as a diagnostic criterion is discussed in detail. Nevertheless, a number of studies have shown associations of three polymorphic variants (single nucleotide polymorphisms, SNPs) in the SELE gene (G98T (rs1805193) in the 5ʹUTR, A561C (rs5361), C1880T (rs5355) in exons 4 and 10, respectively) with severe and subclinical atherosclerosis, coronary heart disease, ischemic heart disease, myocardial infarction, ischemic stroke, Kawasaki disease, and arterial hypertension (Wenzel et al., 1994; Zheng et al., 2001; Yoshida et al., 2003; Zak et al., 2008; Mallik, Majumder, 2011; Shirakawa et al., 2012; Wang Z. et al., 2012; Zhao et al., 2012; Wang X. et al., 2013; Qin et al., 2015; Liao B. et al., 2016; Deng et al., 2017; Vargas-Alarcon et al., 2019; Ding et al., 2021). Thus, associations of exon and promoter polymorphisms of the SELE gene with cardiovascular pathology are shown.

The aim of our study was to investigate the associations of intronic functionally significant polymorphic variants of the SELE gene with the development of ST-elevation myocardial infarction.

## Materials and methods

The study included 74 patients hospitalized in the Department of Emergency Cardiology of the Cardiology Research Institute of the Tomsk NRMC from 2019 to 2021. The diagnosis of primary STEMI was established in accordance with fourth Universal Definition of Myocardial Infarction (Thygesen et al., 2018). The inclusion criteria in the study were: a verified diagnosis of primary STEMI, age over 18 years and a permanent residence in the Tomsk region. The exclusion criteria were: cardiogenic shock, autoimmune, oncologic diseases, terminal chronic kidney disease, atrial fibrillation/atrial flutter, hemodynamically significant valve heart defects, marked cognitive dysfunction. The study protocol adhered to the standards established by the Declaration of Helsinki and received approval from the local ethical committee of the Cardiology Research Institute. A population sample of Russians from Tomsk (136 individuals), formed from DNA samples from the “Biobank of the Population of Northern Eurasia” of the Research Institute of Medical Genetics of the Tomsk NRMC, was used as a control. The groups of patients and the control sample were comparable in sex and age. All examined individuals were ethnically homogeneous and were represented predominantly by Russians (>95 %) from Tomsk: all of them gave informed consent.

Both study groups were predominantly comprised of men, with a male-to-female ratio of 2.1 in the STEMI group and 1.5 in the control group; there were no statistically significant differences between the groups. The mean age in the STEMI group was 61 ± 10 years (median 62.5; interquartile range [55.0–69.0]), and in the control group, 62.1 ± 7 years (median 63.0; interquartile range [57.0–68.0]); there were no statistically significant differences between the groups.

DNA from venous peripheral blood was isolated using the standard phenol-chloroform method (Sambrook, Russell, 2006). Genotyping was performed using real-time polymerase chain reaction (real-time PCR) with the BioMaster HS-qPCR (2×) PCR kit (BioLabMix, Novosibirsk), region-specific primers and TaqMan probes (manufactured by DNA-Synthesis, Moscow) (Table 1).

**Table 1.**
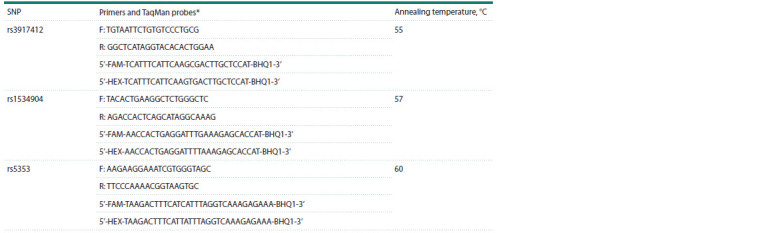
The conditions for marker genotyping in the SELE gene * Primers and samples were selected using the Vector NTI program (http://www.informaxinc.com).

We selected for analysis polymorphic variants in the SELE gene that are eQTL variants for their own or nearby genes (according to GTExPortal (https://www.gtexportal.org/ home/)), potentially having functional significance (according to RegulomeDB (https://regulomedb.org/regulome-search/)), are located in non-coding regions of the gene and have a minor allele frequency of at least 25 % in Caucasians (data from the 1000 Genomes Project, Ensemble (https://www. ensembl.org/index.html)). As a result, three intronic markers were analyzed (Table 1).

Association analysis was performed using standard methods of statistical analysis (χ2, OR with 95 % CI; differences between the compared groups were considered statistically significant at p < 0.05). The method of logistic regression was used to study the inheritance model. Linkage analysis (including calculation of the linkage disequilibrium coefficient (Dʹ)) was performed in the Haploview 4.2 program (Barrett et al., 2005).

The functional annotation of the variants was performed using the VannoPortal resource (http://www.mulinlab.org/ vportal/index.html/). In particular, the PhyloP, GerpN, and GerpS scores are provided to assess evolutionary conserva tism. The PhyloP score is used to estimate evolutionary conservatism based on interspecies comparisons, with humans excluded from the analysis (the prefixes denote classification ranks, in this case: pri – primates) (Pollard et al., 2010; Caron et al., 2019). The GerpN and GerpS scores are based on single nucleotide analysis, namely the analysis of the homology of the locus across species (GerpN) and the analysis of the deficit or surplus in substitutions at the locus (GerpS) (Zerbino et al., 2018; Caron et al., 2019). The Phred Score is a measure of the quality of the score obtained (if Phred assigns a quality score of 20, the error probability is 1 %, score of 10 means an error probability of 10 %). To evaluate the selection effect, we use data from Neilsen’s CLR (Composite Likelihood Rate) test – Composite Likelihood method to determine the strength of positive selection (Vy, Kim, 2015).

Protein-protein interactions were evaluated using the BioGRID resource (https://thebiogrid.org/) (Oughtred et al., 2021). Functional enrichment analysis was performed using WebGestalt (WEB-based GEne SeT AnaLysis Toolkit) (https://www.webgestalt.org/) (Liao Y. et al., 2019).

## Results

Genotyping of three intronic variants (rs5353, rs1534904, rs3917412) in the SELE gene was performed in the population sample of Tomsk and in the group of STEMI patients.

Linkage and association analysis

We revealed the complete linkage of the rs5353 marker with two other SNPs studied (Dʹ = 1 in both patients and control sample). In turn, rs3917412 and rs1534904 are closely but not completely linked (Dʹ is 0.916 in the patient group and 0.976 in the population sample). The linkage analysis of the studied markers indicates that the substitution in rs5353 occurred within the context of the haplotype comprising the reference rs3917412 and rs1534904 alleles. As a result, we expect a multidirectional effect of the studied nucleotide substitutions (rs5353 on the one side, rs3917412 and rs1534904 on the other side) on the manifestation of pathological features

We found statistically significant differences in the frequencies of rs5353 genotypes in the SELE gene between patients and the control sample ( p = 0.004) (Table 2). According to logistic regression (Table 3), two models, codominant and recessive, were statistically significant. However, the information criteria (Akaike and Bayesian) are the lowest for the recessive model, which defines it as the best model. Consequently, the CC genotype was identified as a risk factor for myocardial infarction, occurring six times more frequently in the patient group (OR = 6.93; CI:95 % (1.84–26.04); χ2 = 8.69; p = 0.002) (Tables 2 and 3).

**Table 2.**
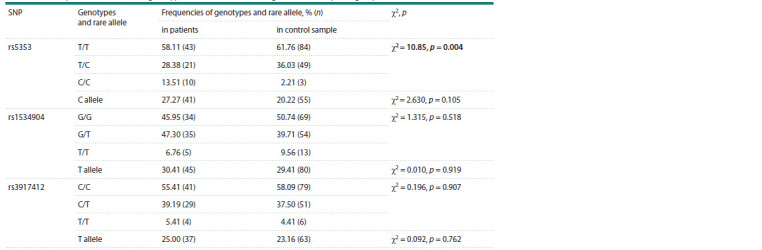
The frequencies of alleles and genotypes of markers in the SELE gene in the compared groups Notе. Statistically significant differences are highlighted in bold.

**Table 3.**
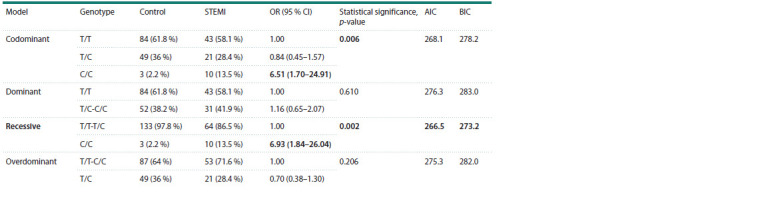
Models of predisposing effect inheritance of rs5353 in the SELE gene Notе. Statistically significant differences are highlighted in bold.

The studied samples exhibited statistically significant differences in the genotype combination (χ2 = 22.76; df = 8; p = 0.004). The observed differences can be attributed to two distinct combinations of genotypes (rs5353/rs1534904/ rs3917412): CC/GG/CC predisposes to the development of myocardial infarction (OR = 6.93, CI:95 % (1.68–32.98), χ2 = 8.69, p = 0.003), whereas the TC/GG/CC combination is protective (OR = 0.38, CI:95 % (0.16–0.90), χ2 = 5.01, p = 0.02). So, the CGC haplotype is more prevalent in patients than in controls (27.7 % and 20.5 %, respectively) and the TGC haplotype is more prevalent in controls (40.4 % in patients and 49.2 % in controls), but these differences are not statistically significant

In the sample of patients, a deviation from Hardy–Weinberg equilibrium was identified ( p = 0.012). There is a deficiency in heterozygotes and frequent allele homozygotes, but an excess of rare homozygotes. The quality control of genotyping (100 % re-genotyping of the patient sample) confirmed the correctness of the experiment. We can conclude that in this case there is a biological reason for the deviation from the Hardy–Weinberg equilibrium, given the a priori bias in the patient sample and the prevalence of the pathology-associated genotype. On the other hand, the studied sample of patients is not large, and thus the results obtained require further validation on larger samples.

Functional analysis of the studied markers

The studied markers are located in introns 1 (rs5353), 4 (rs1534904) and 5 (rs3917412) of the SELE gene. Notably, the degree of conservatism of the studied markers exhibits considerable variability. So, rs5353 is conservative only in primates (Phred Score = 11.93 for priPhyloP), rs1534904 is probably conservative not only in primates (Phred Score = 16.10 for priPhyloP), but also in various species in general (Phred Score = 16.58 and 11.11 for GerpN and GerpS, respectively), rs3917412 is not conservative (according to VannoPortal resource). All the studied substitutions are under positive selection (Neilsev’s CLR test), according to information from the VannoPortal resource.

According to the RegulomeDB classification, all three of the studied markers belong to functional class 1f, i. e. they are eQTL variants in the transcription factor (TF) binding motif or in the DNAase hypersensitivity region. Indeed, the studied substitutions can theoretically (i. e. according to bioinformatic analysis) alter the affinity of a number of TFs (according to the VannoPortal resource). Thus, 24 TF binding sites have been identified for rs5353, with 17 of them being lost in the presence of an alternative allele. Nevertheless, according to experimental studies, physical interactions of these TFs with DNA in this region have not yet been detected in the studied tissues. Further seven substitutions resulted in the formation of novel transcription factor binding sites (ATF1, CEBPA, HOXA1, JUND, REST, JUNB) (Table 4). In the case of rs1534904, there are 11 theoretically variable sites, of which four are lost (not actually detected) in the presence of the alternative allele, but seven new sites emerge (PRDM1, RORC, IRF5, ZNF143, NCOR1, ZEB1, POU2F2) (Table 4). For rs3917412, theoretical calculations indicate the presence of 16 TF binding sites; the alternative allele results in the emergence of two novel sites (CHD2, PAX1) (Table 4), the disappearance of ten sites, and a decrease in affinity of four TFs. As in the previous cases, the list of theoretically variable TFs does not include physically detected interactions (according to the VannoPortal resource).

**Table 4.**
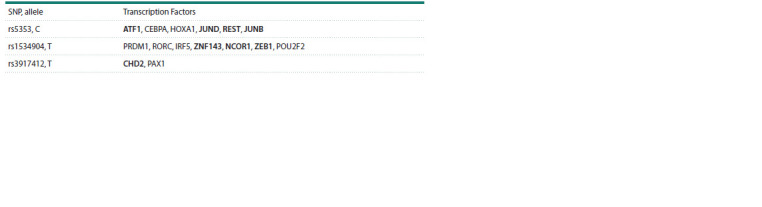
Transcription factors for which binding sites emerge in the presence of rare alleles of the studied markers Notе. TFs expressed in vascular walls are highlighted in bold (compiled according to the VannoPortal and GTEx Portal resources).

As eQTL variants, the studied markers are associated with changes in the expression levels of SELE and nearby genes (see the Figure); in addition, rs1534904 alters the splicing site of the C1orf112 (FIRRM) gene (according to the GTEx Portal resource). The effect of the studied nucleotide substitutions can therefore be realized not only through the biochemical pathways involving E-selectin, but also through pathways involving other regulated genes. To date, six regulated genes have been identified for rs5353 and rs3917412 and five for rs1534904; a total of nine genes (FIRRM, KIFAP3, METTL18, RN7SL333P, RP1-206D15.6, RP1-117P20.3, SCYL3, SELE, SELL) have been identified (GTEx Portal). The FIRRM and KIFAP3 genes seem to be the most interesting, as the effect of the examined substitutions in the SELE gene on their expression corresponds with the linkage outcomes: while KIFAP3 expression is reduced in homozygotes for the rare allele rs5353, it is elevated in homozygotes for the rs1534904 and rs3917412 derived alleles. Conversely, FIRRM expression is lower in carriers of the rs1534904 and rs3917412 derived alleles, but higher in carriers of the rare rs5353 allele (see the Figure).

**Fig. 1.**
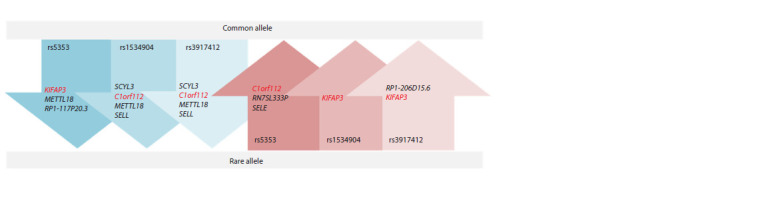
Scheme of alterations in the expression levels of a number of genes depending on rs5353, rs1534904, rs3917412 in the SELE gene. Compiled according to the GTEx Portal resource.

In light of these observations, three proteins appear to be the most promising in terms of realizing the functional effect of the studied substitutions. For E-selectin, a small number of interactors are detected: there are only 19 interacting molecules including two chemical compounds and 17 proteins (according to the BioGRID resource). The WebGestalt enrichment analysis indicates that the most significant processes, with the largest number of interactor proteins involved, are hemostasis (10 proteins, padj = 6.4E–7), platelet activation, signaling and aggregation (6 proteins, padj = 3.6E–4) and cell surface interactions at the vascular wall (5 proteins, padj = 3.6E–4). The results are in complete alignment with the known biochemical functions of E-selectin. For FIGNL1 Interacting Regulator of Recombination and Mitosis encoded by the FIRRM (C1orf112) gene, 60 interactor proteins are identified (according to the BioGRID resource). The most significant processes with the largest number of interactors involved include serpentine receptor ligand binding (19 proteins, padj = 3.8E–13) and various types of signaling through these receptors (16–20 proteins each, padj = 4.5E–12–4.2E–7) (according to WebGestalt). The protein product of the FIRRM gene has not yet been sufficiently studied, but in addition to its originally described role as a kinetochore protein (Xu et al., 2021), it is also known to be involved in DNA repair processes (Mazouzi et al., 2023; Pinedo-Carpio et al., 2023; Tischler et al., 2024). A total of 105 interactor proteins have been identified for the KIFAP3 gene product (kinesin-2 associated protein) according to the BioGRID resource. The results of the enrichment analysis indicate that the most significant processes with the highest number of interactors involved (by WebGestalt) are the adaptive immune response (16 proteins, padj = 6.2E–3), different mRNA processing pathways (7–9 proteins, padj = 6.2E–3–6.4E–3), and antigen presentation via major histocompatibility complex class II (7 proteins, padj = 6.2E–3). These processes have the highest number of interactors involved, as determined by WebGestalt.

## Discussion

Thus, all three proteins encoded by the SELE, FIRRM, and KIFAP3 genes (co-regulated by the studied intronic variants) are not directly involved in the development of cardiovascular events but may be indirectly involved in the pathogenesis of CVDs through the inflammation, immune response, and DNA repair systems

Of the nine genes co-regulated by the studied markers, four genes showed no associations with pathologies or quantitative traits identified by GWAS. Three of them do not encode proteins: two genes of long non-coding RNAs (RP1-206D15.6, RP1-117P20.3) and a pseudogene (RN7SL333P); and one gene (METTL18) encodes a methyltransferase. For the remaining five genes, GWAS demonstrated a multitude of associations (Supplementary Material, compiled from GWAS catalog)1. Three of them (FIRRM, KIFAP3, SELE) are discussed in detail above; the SELL gene encodes L-selectin, which promotes leukocyte rolling as well as E-selectin (GeneCard, https://www. genecards.org/); the SCYL3 gene encodes a pseudokinase that also plays a role in cell adhesion and migration (GeneCard, https://www.genecards.org/). Markers in the region of location of these five genes showed associations with various blood biochemical parameters, cell composition of blood (FIRRM, KIFAP3, SCYL3, SELE, SELL), as well as with amyotrophic lateral sclerosis and venous thromboembolism (FIRRM, KIFAP3), type 2 diabetes mellitus (FIRRM, SELL) (Supplementary Material). We would like to emphasize that among the 64 SNPs associated with various pathologies or quantitative traits according to GWAS results, there are neither the markers studied in the present study (rs5353, rs1534904 and rs3917412), nor the SNPs associated with cardiovascular diseases according to earlier studies (rs1805193, rs5361, rs5355).


Supplementary Materials are available in the online version of the paper: https://vavilov.elpub.ru/jour/manager/files/Suppl_Babush_Engl_29_1.pdf


It should be noted that the polymorphic variants analyzed in this study had not been previously studied in the context of CVDs and had seldom been included in associative studies (there is no information on these markers in the PubMed, DisGeNet, GWAS Catalog databases). Only one study has been found in the available literature that has demonstrated the risk effect of the GG rs3917412 genotype on the development of colon cancer (Custodio et al., 2014). At the same time, genomic estimates of pathogenicity (which reflect the probability of marker involvement in the development of multifactorial pathology, estimated by regBase (Zhang S. et al., 2019)) indicate that the analyzed variants may be involved in pathological processes. Additionally, the oncogenicity estimates indicate the probability of a “driver” effect (likely cancer driver) of these nucleotide substitutions for the development of oncopathology (according to the VannoPortal resource).

## Conclusion

Thus, the mechanism of E-selectin involvement in the pathogenesis of STEMI is not fully understood. On the one hand, a sufficient amount of biochemical data indicates its involvement in the development of CVDs (Liao B. et al., 2016; Deng et al., 2017; Vargas-Alarcon et al., 2019; Ding et al., 2021), and primarily in the development of such IHD risk factors as atherosclerosis and DM2 (Roldán et al., 2003; McEver, 2015; Qiu et al., 2019; Mathur et al., 2023). On the other hand, the involvement of this protein in the inflammatory response suggests its involvement primarily in the recovery processes after a cardiovascular event, and only indirectly in the development of susceptibility to myocardial infarction (Ueno, 2012; Sandoval-Pinto et al., 2014; Srivastava et al., 2018). The association of rs5353 with STEMI revealed in the present study provides further confirmation of the involvement of the SELE gene in the development of CVDs. Additionally, the analysis demonstrates the presence of a region more extensive than one gene, which is co-regulated by the nucleotide substitutions studied. It is possible that this entire genome region may be involved in the pathogenesis of CVD indirectly, through the inflammation, immune response, and DNA repair systems.

## Conflict of interest

The authors declare no conflict of interest.
